# Cortical thickness in Parkinson disease: A coordinate-based meta-analysis

**DOI:** 10.1097/MD.0000000000021403

**Published:** 2020-07-31

**Authors:** LiQin Sheng, PanWen Zhao, HaiRong Ma, Joaquim Radua, ZhongQuan Yi, YuanYuan Shi, JianGuo Zhong, ZhenYu Dai, PingLei Pan

**Affiliations:** a Department of Neurology, Kunshan Hospital of Traditional Chinese Medicine, Kunshan; b Department of Central Laboratory; c Imaging of Mood- and Anxiety-Related Disorders (IMARD) group, Institut d’Investigacions Biomèdiques August Pi i Sunyer (IDIBAPS), Centro de Investigación Biomèdica en Red de Salud Mental, Barcelona, Spain; d Early Psychosis: Interventions and Clinical-detection (EPIC) Lab, Department of Psychosis Studies, Institute of Psychiatry, Psychology and Neuroscience, King's College London, London, UK; e Department of Clinical Neuroscience, Centre for Psychiatric Research and Education, Karolinska Institutet, Stockholm, Sweden; f Department of Neurology; g Department of Radiology, Affiliated Yancheng Hospital, School of Medicine, Southeast University, Yancheng, P.R. China.

**Keywords:** coordinate-based meta-analysis, cortical thickness, gray matter, Parkinson disease, Seed-based *d* Mapping, surface-based morphometry

## Abstract

**Background:**

A growing number of studies have used surface-based morphometry (SBM) analyses to investigate gray matter cortical thickness (CTh) abnormalities in Parkinson disease (PD). However, the results across studies are inconsistent and have not been systematically reviewed. A clear picture of CTh alterations in PD remains lacked. Coordinate-based meta-analysis (CBMA) is a powerful tool to quantitatively integrate the results of individual voxel-based neuroimaging studies to identify the functional or structural neural substrates of particular neuropsychiatric disorders. Recently, CBMA has been updated for integrating SBM studies.

**Methods:**

The online databases PubMed, Embase, Web of Science, China National Knowledge Infrastructure (CNKI), WanFang, and SinoMed were comprehensively searched without language limitations from the database inception to February 2, 2020. We will include all SBM studies that compared regional CTh between patients with idiopathic PD and healthy control subjects at the whole-cortex level using Seed-based *d* Mapping with Permutation of Subject Images (SDM-PSI). In addition to the main CBMA, we will conduct several supplementary analyses to test the robustness of the results, such as jackknife analyses, subgroup analyses, heterogeneity analyses, publication bias analyses, and meta-regression analyses.

**Results:**

This CBMA will offer the latest evidence of CTh alterations in PD.

**Conclusions:**

Consistent and robust evidence of CTh alterations will feature brain morphometry of PD and may facilitate biomarker development.

**PROSPERO registration number:**

CRD42020148775

## 1 Introduction

Parkinson disease (PD) is a common neurodegenerative disease^[1]^ that affected 6.1 million individuals worldwide.^[2,3]^ PD is a highly clinically heterogeneous condition traditionally characterized by cardinal motor symptoms, such as resting tremor, rigidity, and bradykinesia; however, PD also manifests various non-motor symptoms throughout the course of disease, such as cognitive impairment, apathy, and depression.^[4,5]^ The neurophysiology of PD is complex. Modern neuroimaging techniques have featured prominently in attempts to understand the pathophysiology in vivo.^[6–9]^

Cortical thickness (CTh) analysis is popular surface-based technique for cortical gray matter (GM) assessment. A growing number of studies have used CTh analysis to investigated brain morphology in PD relating to demographic and clinical characteristics, such as age of onset, age, disease duration, motor deficits, disease stages, and divergent non-motor symptoms.^[10–38]^ Some studies suggested that CTh alterations may be indicators of neural degeneration occurring in PD,^[39,40]^ while some other CTh studies argued against that view as they failed to identify morphological features in patients with PD relative to healthy controls (HCs).^[31,41–44]^ Despite the many advances in our understanding of the neurobiological underpinnings of PD, the results of the CTh analysis in PD across studies are inconsistent and have not been systematically reviewed.

Coordinate-based meta-analysis (CBMA) is powerful tool to quantitatively integrate the results of individual neuroimaging studies.^[45,46]^ Recently, CBMA has been developed for surface-based morphometric (SBM) studies to identify consistent CTh abnormalities in major depressive disorder.^[47]^ In the present study, we conducted a CBMA of SBM studies that investigate CTh alterations in PD using Seed-based *d* Mapping with Permutation of Subject Images (SDM-PSI).^[48,49]^

## 2 Methods

This protocol will follow the Preferred Reporting Items for Systematic review and Meta-Analysis Protocols (PRISMA-P).^[50]^ The protocol of this CBMA was registered at PROSPERO (http://www.crd.york.ac.uk/PROSPERO) (registration number: CRD42020148775). No ethical approval is required because the data used in this paper are from published studies without the involvement of animals or individual experiments.

### 2.1 Data sources and study selection

The online databases PubMed, Embase, and Web of Science will be searched, using the keywords

((Parkinson disease) or Parkinson∗) and ((cortical Thickness) OR (cortical thinning) OR (surface-based morphometry)) without language limitations from the database inception to the July 1, 2019 and updated on Feb 2, 2020. The following databases China National Knowledge Infrastructure (CNKI), WanFang, and SinoMed will be also searched for studies published in Chinese. Additionally, the reference lists of relevant reviews and the articles selected for inclusion were further manually searched.

### 2.2 Eligibility criteria

#### 2.2.1 Inclusion criteria

The articles included in the CBMA should meet the following inclusion criteria: included patients with idiopathic PD diagnosed according to the accepted criteria; compared regional CTh differences between patients with idiopathic PD and HC subjects at the whole-brain cortical level; reported peak coordinates of significant clusters in standard Montreal Neurological Institute (MNI) or Talairach space; was a case-control original article published in a peer-reviewed journal without the limit of language.

#### 2.2.2 Exclusion criteria

Publications will be excluded if they met the following exclusion criteria: the sample size was fewer than 7 either in the PD group or the HC group^[45]^; the studies included PD patients with dementia; the studies reported significant results without listing three-dimensional coordinates; the studies only employed regions of interest (ROI) analysis; the studies only conducted the global CTh analysis; the studies lacked an HC group; the studies with the patient samples were overlapped with the another one with the largest sample size. The studies were longitudinal without performing baseline comparisons. The publications were not an original type, such as conference abstracts, research protocols, letters, reviews, and editorials.

**Figure 1:**
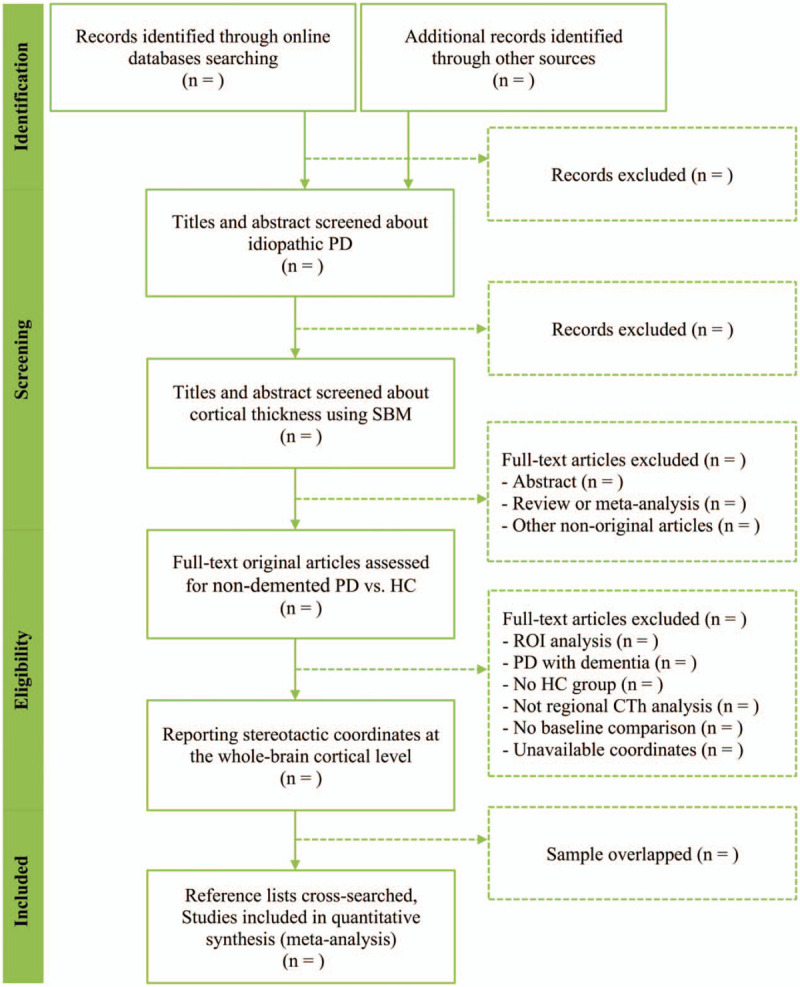
Study selection process in accordance with the PRISMA flowchart. CTh = cortical thickness; PD = Parkinson's disease; PRISMA = Preferred Reporting Items of Systematic Review and Meta-Analysis, ROI = regions of interest; SBM = surface-based morphometry.

Figure 1 presents the process of study selection in accordance with the PRISMA flowchart.

### 2.3 Quality assessment

Currently, there was no objective tool to perform quality assessment for CTh studies. Referring to the previous work,^[47]^ we used a 12-point checklist to assess the quality of each included study in the CBMA (Table 1). This checklist integrated the items regarding the sample characteristics and imaging-specific methodology employed in the studies.

**Table 1:**
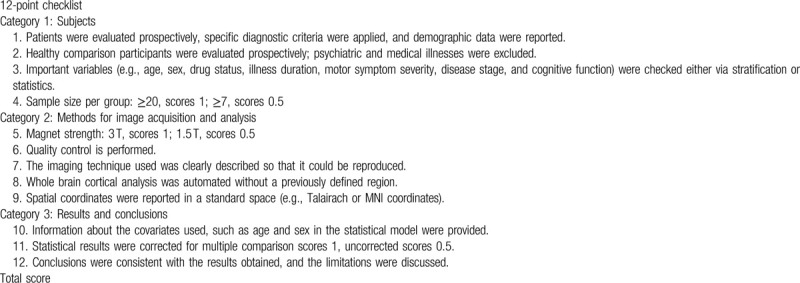
The checklist of quality assessment for the included cortical thickness studies.

### 2.4 Data extraction

The following specific data will be extracted from the included studies: the first author's family name, publication year, sample size, male number, age, education (years), disease duration, United Parkinson disease rating scale, part III (UPDRS-III), Hoehn and Yahr (HY) stage, mini-mental state examination (MMSE), levodopa equivalent daily dose (LEDD), MRI scanner manufacturer and platform, field strength, head coil, MRI sequence, voxel size, imaging processing software package, smooth kernel, statistical model, covariate, statistical threshold, peak coordinates, height of the peaks (*t* value or *z*-value, but a *P*-value is also useful), and their stereotactic reference space.

Two of the authors (LQS and PWZ) will independently perform the literature search, study selection, quality assessment, and data extraction. Any inconsistencies will be discussed by consensus.

### 2.5 Data analysis

#### 2.5.1 Main CBMA

The main CBMA will be carried out using the SDM-PSI software package (version 6.21, https://www.sdmproject.com/). Its standard procedures include: calculation of the maps of the lower and upper bounds of possible effect sizes for each study separately based on the peak information using a specific FreeSurfer GM mask, full anisotropy = 1, isotropic full width half maximum = 20 mm, and voxel = 2 mm; the mean analysis: estimation of the map of most likely effect size and its standard error based on the MetaNSUE algorithms,^[51,52]^ conducting multiple imputations of the maps of effect size of the individual studies, meta-analysis of these maps using a standard random-effects model, and Rubin rules to pool the different meta-analyses resulting from the multiple imputations^[52]^; family-wise error (FWE) correction for multiple comparisons using common permutation tests; and finally use of threshold-free cluster enhancement (TFCE) in the statistical thresholding (*P* < .05, voxel extent ≥10). The details of these procedures have been extensively described in prior publications^[48,49]^ and the SDM-PSI reference manual (https://www.sdmproject.com/manual/).

#### 2.5.2 Subgroup CBMA

Subgroup CBMA will be conducted when the number of the datasets is sufficient (n ≥ 10). Subgroup CBMA would be performed in clinical subtypes (such as PD patients without mild cognitive impairment and patients with mild cognitive impairment) and imaging methodology variables (including the datasets using 3.0 Tesla MRI scanners, slice thickness lower than 1 mm or voxel size lower than 1 × 1 × 1 mm^3^, FreeSurfer software packages, FWHM of the smoothing kernel size of ≤15 mm, at least 1 covariates included in the statistical model, and thresholds corrected for multiple comparisons as well as those datasets performing quality control for imaging data and those not specifying it).

#### 2.5.3 Jackknife sensitivity, heterogeneity, and publication bias analyses

To test the influence of each dataset on the pooled CBMA results, Jackknife sensitivity analysis will be performed by iteratively repeating the same analysis *K* – 1 times (*K* = the number of datasets included), discarding one dataset each time.^[53,54]^

Where there is a significant cluster with a peak MNI coordinate reported in the CBMA, we will extract the information to derive standard heterogeneity statistics *I*^2^, with *I*^2^ < 50% indicating low heterogeneity.

Publication bias of the significant cluster will be assessed with a test analogue to the Egger test (*P* < .05).

#### 2.5.4 Meta-regression analysis

Meta-regression analyses will be carried out to examine the potential effects of age, male sex, disease duration, UPDRS-III, HY stage, MMSE, and LEDD on the CBMA results. Statistical significance will be determined using the TFCE-based FWE corrected threshold (*P* < .05, voxel extent ≥10).^[48,49]^

## 3 Discussion

To our best knowledge, this is the first CBMA of SBM studies to quantitatively identify consistent CTh alterations in PD using the latest algorisms of SDM-PSI^[48,49]^ and following the recent guidelines and recommendations.^[45,46]^ In addition to the main CBMA, we will conduct several supplementary analyses to test the robustness of the results, such as jackknife analyses, subgroup CBMA analyses, heterogeneity analyses, publication bias analyses, and meta-regression analyses. This CBMA will provide the latest evidence of CTh alterations in PD.

PD is a progressive neurodegenerative disorder. It has been suggested that cortical neurodegeneration emerged at stage 4 (associated with early phase motor dysfunction) and later stages according to the well-established brain pathologic staging scheme for PD proposed by Braak et al.^[55]^ All the datasets included in the current CBMA enrolled patients with PD at their symptomatic stages, which indicate that cortical neurodegeneration should exist probably manifesting CTh alterations. If there was statistically significant consistence of CTh alterations with robustness in PD, we would discuss the importance of such findings involved in the pathophysiological mechanisms. If not, we would discuss the potential confounding factors the lead to this lack of consistence, such as small sample sizes, sample heterogeneity, and imaging methodological variations. We would further propose some recommendations to design robust CTh studies.

## Author contributions

**Conceptualization:** JianGuo Zhong, ZhenYu Dai, PingLei Pan.

**Data curation:** PanWen Zhao, ZhongQuan Yi.

**Formal analysis:** HaiRong Ma, LiQin Sheng.

**Funding acquisition:** PingLei Pan.

**Investigation:** LiQin Sheng, PanWen Zhao, ZhongQuan Yi.

**Methodology:** Joaquim Radua, LiQin Sheng, PingLei Pan.

**Project administration:** LiQin Sheng, PingLei Pan.

**Resources:** PanWen Zhao, ZhongQuan Yi.

**Software:** Joaquim Radua, LiQin Sheng.

**Supervision:** JianGuo Zhong, ZhenYu Dai, PingLei Pan.

**Validation:** PingLei Pan.

**Visualization:** HaiRong Ma, LiQin Sheng.

**Writing – original draft:** LiQin Sheng, PanWen Zhao, HaiRong Ma.

**Writing – review & editing:** Joaquim Radua, JianGuo Zhong, ZhenYu Dai, PingLei Pan.
